# Cost-Effectiveness of Saxagliptin versus Acarbose as Second-Line Therapy in Type 2 Diabetes in China

**DOI:** 10.1371/journal.pone.0167190

**Published:** 2016-11-22

**Authors:** Shuyan Gu, Yuhang Zeng, Demin Yu, Xiaoqian Hu, Hengjin Dong

**Affiliations:** 1 Center for Health Policy Studies, School of Public Health, Zhejiang University School of Medicine, Hangzhou City, Zhejiang Province, China; 2 Key Laboratory of Hormones and Development (Ministry of Health), Metabolic Diseases Hospital and Tianjin Institute of Endocrinology, Tianjin Medical University, Tianjin, China; Shanghai Diabetes Institute, CHINA

## Abstract

**Objective:**

This study assessed the long-term cost-effectiveness of saxagliptin+metformin (SAXA+MET) versus acarbose+metformin (ACAR+MET) in Chinese patients with type 2 diabetes mellitus (T2DM) inadequately controlled on MET alone.

**Methods:**

Systematic literature reviews were performed to identify studies directly comparing SAXA+MET versus ACAR+MET, and to obtain diabetes-related events costs which were modified by hospital surveys. A Cardiff Diabetes Model was used to estimate the long-term economic and health treatment consequences in patients with T2DM. Costs (2014 Chinese yuan) were calculated from the payer’s perspective and estimated over a patient’s lifetime.

**Results:**

SAXA+MET predicted lower incidences of most cardiovascular events, hypoglycemia events and fatal events, and decreased total costs compared with ACAR+MET. For an individual patient, the quality-adjusted life-years (QALYs) gained with SAXA+MET was 0.48 more than ACAR+MET at a cost saving of ¥18,736, which resulted in a cost saving of ¥38,640 per QALY gained for SAXA+MET versus ACAR+MET. Results were robust across various univariate and probabilistic sensitivity analyses.

**Conclusion:**

SAXA+MET is a cost-effective treatment alternative compared with ACAR+MET for patients with T2DM in China, with a little QALYs gain and lower costs. SAXA is an effective, well-tolerated drug with a low incidence of adverse events and ease of administration; it is anticipated to be an effective second-line therapy for T2DM treatment.

## Introduction

Diabetes mellitus is a huge and growing health problem in the 21st century, and the costs to healthcare systems and society are high and escalating. It is estimated by the International Diabetes Federation that 387 million people worldwide had diabetes in 2014; by 2035, this number is expected to rise to 592 million [1]. Adult diabetics in China and India are predicted to account for almost a third of the world’s diabetic population by the year 2025 [2]. The number of people with type 2 diabetes mellitus (T2DM) is increasing worldwide [1]. In China in 2014, there were approximately 96 million adult diabetics (20−79 years of age), with a 9.32% national diabetes prevalence and 51 million undiagnosed diabetes cases [1]. Meanwhile, diabetes management is poor in China; only 25.8% of patients receive diabetes treatment and only 39.7% of those treated achieve adequate blood glucose control [3]. As a result, diabetes caused 1.2 million deaths in China in 2014, which accounted for 24.6% of the world’s diabetes-related deaths [1].

In light of poor management and high numbers of diabetes-related deaths, a series of antidiabetic drugs have been successively introduced into the Chinese pharmaceutical market in recent decades. Despite the proven efficacy of these antidiabetic drugs, their nonnegligible adverse effects (hypoglycemia with sulfonylureas [SUs] and insulin; weight gain with insulin, SUs, and thiazolidinediones; gastrointestinal [GI] discomfort with metformin [MET], α-glucosidase inhibitors [AGIs], and glucagon-like peptide-1 [GLP-1] receptor agonists) [4–6] have impacted patient compliance, further impeded treatment effects, and elevated healthcare costs [7–11].

Patient compliance is a major problem in T2DM treatment. Negligible or suboptimal adherence to diabetes medications is associated with poor blood glucose control, increased risk of hospitalization, and mortality [12–16]. Conversely, higher medication adherence can improve health outcomes, limit the development of complications, and lower healthcare resource utilization and costs for patients [8,11,17–18]. Efficacy and tolerability are no longer the only criteria used to assess a drug; ease of administration, convenient dosing frequency, and favorable adverse event profiles that may lead to better patient compliance are also essential factors [8,19–21]. Therefore, new antidiabetic drugs with proven efficacy and favorable adverse event profiles, as well as ease of administration are clearly needed to better address this unmet need, with the goal of enhancing patient quality of life (QOL) and lifespan.

Acarbose (ACAR) and saxagliptin (SAXA) are both recommended as second-line therapies for T2DM treatment in China [5]. ACAR, an AGI, acts by competitively inhibiting the digestion and absorption of carbohydrates in the small intestine, reducing the increase in blood glucose concentrations after a carbohydrate load [5,22]. ACAR lowers postprandial glucose levels without causing hypoglycemia and malabsorption, is generally safe and well tolerated [23–25], and may provide beneficial cardiovascular outcomes for patients with T2DM [22,26]. However, these benefits may be offset by its non-negligible GI adverse events, frequent dosing schedule (3 times/d) and inconvenient administration (should be chewed with the first mouthful of food, or swallowed whole with a little liquid directly before the meal) [27], which can limit long-term patient compliance with therapy [6,21,24–25].

Saxagliptin is a new dipeptidyl peptidase-4 inhibitor (DPP-4i) that inhibits the breakdown of the incretin hormones GLP-1 and glucose-dependent insulinotropic polypeptide, resulting in increased glucose-dependent insulin secretion and suppression of glucagon secretion [28]. As monotherapy or in combination regimens, SAXA consistently improves blood glucose control in patients with T2DM by effectively lowering glycated hemoglobin (HbA1c) levels and fasting plasma and postprandial glucose and is generally safe and well tolerated. Additionally, it confers a low risk of hypoglycemia, weight gain, and cardiovascular events [28–31] and provides the additional advantages of fewer GI adverse events, convenient dosing frequency (1 time/d) and ease of administration (can be taken with or without a meal at any time of the day) [32] compared with ACAR, which may lead to better patient compliance [21,33].

Choice of antidiabetic drugs should evaluate individual patient characteristics, preferences, and values and balance the need to optimize blood glucose control with the need to minimize adverse events [6]. As an effective drug with favorable adverse event and weight-loss profiles, SAXA is a promising option as second-line therapy compared with ACAR, although its high cost may be a barrier to widespread use [4,6].

To our knowledge, there are no Chinese or international studies that directly compare both long-term benefit and cost aspects of SAXA with ACAR as second-line add-on therapy to MET, and there are only few short-term head-to-head Chinese clinical trials comparing the efficacy of SAXA with ACAR as an add-on to MET identified. From the health insurance payer’s perspective, this study aims to estimate the long-term cost-effectiveness of SAXA+MET compared with ACAR+MET in T2DM patients with glucose inadequately controlled on MET alone in China.

## Methods

### Cost-effectiveness model

We used a previously published and validated simulation model, the Cardiff Diabetes Model, which is designed to estimate the long-term economic and health impact of comparable medical therapies in patients with diabetes [34–36]. The model is able to run in two key modes (mean values analysis and probabilistic sensitivity analysis) and allows to perform univariate sensitivity analyses by using its inside Tornado model. The model is a patient level fixed-time increment simulation model, which used the equations from the UK Prospective Diabetes Study (UKPDS) 68 to simulate disease progression and forecast the incidence of diabetes-related complications (i.e., microvascular and macrovascular events), mortality and cost-effectiveness in the simulated population [37]. We simulated a cohort of 1000 individuals with T2DM over a 40-year lifetime horizon (mean baseline age: 44 years) [38]. At the beginning, a patient cohort is generated based on the baseline demographics, clinical and modifiable risk factor profiles. Modifiable risk factors are adjusted to reflect any treatment effect specified for HbA1c, weight, cholesterol and/or systolic blood pressure (SBP), and progressed in line with estimations of their natural history. Each simulated subject is then progressed through the model in 6-monthly time increments. Each risk factor influences the incidence of clinical events and thus alters the probability of events over time. Once the trajectories of risk factors are updated, checks are made for specific fatal or non-fatal events. The simulation terminated at patient death or arrival at the time horizon, and all costs and quality adjusted life years (QALYs) are accumulated for that subject. The model then begins simulation for next subject. Once all subjects are simulated the process ends and all summary statistics are collected. The cost-effectiveness result was assessed in terms of the incremental cost-effectiveness ratio (ICER) (i.e., incremental cost per QALY gained), and costs and QALYs associated with each therapy were calculated from the payer’s perspective. Annual discount rates for both costs and benefits were 3% according to the World Health Organization (WHO) guideline [39].

Hypoglycemia and other adverse events were modeled depending on therapy-specific incidence rates. Hypoglycemia was separated into symptomatic and severe events in the model, and severe events (defined as a severe impairment in consciousness requiring medical assistance) were correlated with healthcare costs [5,40]. The model also allowed for customization of adverse events in each therapy arm. For each hypoglycemia event and adverse event, the model evaluated the probability of that event occurring and the associated cost and disutility.

### Literature review and hospital survey

A literature review was conducted to evaluate the arts of the disease state and to collect patient profiles, clinical data for each target treatment, and relevant costs for diabetes-related events. A series of English-language databases (PubMed, Web of Knowledge [including Web of Science, MEDLINE, BIOSIS Citation Index, Derwent Innovations Index], Cochrane Library and ScienceDirect) and Chinese-language databases (China National Knowledge Infrastructure, Chongqing VIP, and Wanfang Data) were systematically searched for relevant studies dating from "2009/01/01" to "2016/09/30" (Date of first authorization of saxagliptin in USA: 1st October 2009) [32] that provided head-to-head comparison of SAXA+MET versus ACAR+MET for patients with T2DM who were inadequately controlled on MET alone.

**Fig 1:**
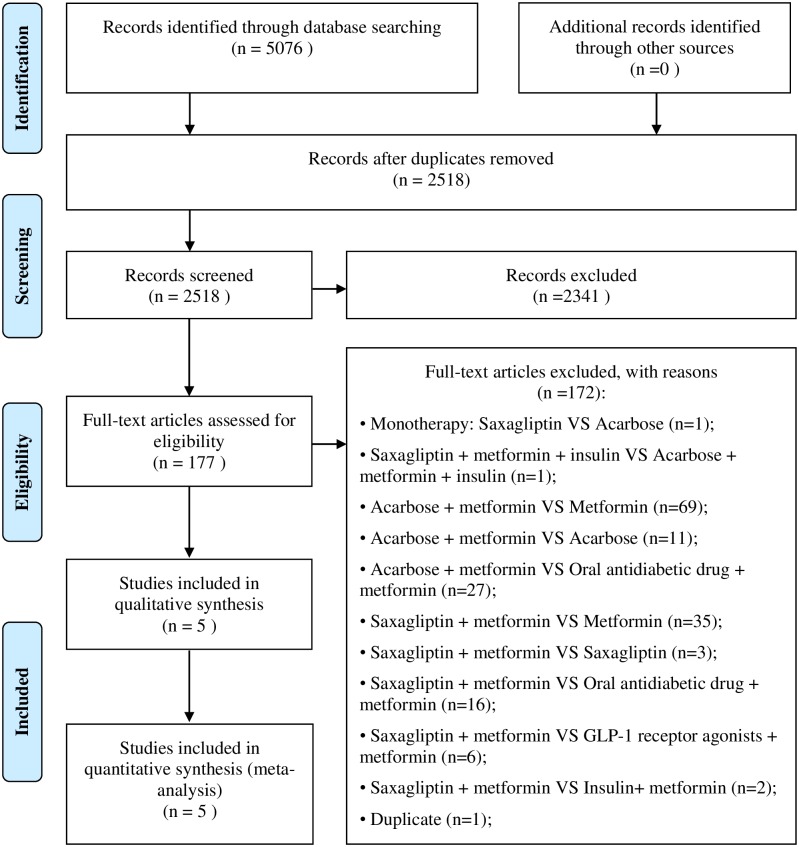
Flow diagram of literature review. A detailed flow diagram that depicts search and selection processes.

Search terms included “saxagliptin”, “Onglyza”, “acarbose” or “Glucobay”, in combination with “type 2 diabetes”, “non-insulin-dependent diabetes mellitus” or “T2DM”, and “Chinese” or “China” (Detailed search strategies are provided in S1 Table). Inclusion criteria were set as follows: (1) Adult Chinese patients with T2DM older than 18 years, (2) study duration up to 12 weeks, (3) randomized controlled trials (RCTs) that head-to-head compared the effect of SAXA+MET versus ACAR+MET, (4) only diet and/or exercise allowed for patients except those receiving target drugs, and (5) appropriate clinical efficacy data to ensure a full review (Detailed selection criteria are provided in S1 Appendix). Two reviewers independently evaluated the search results and extracted the data. A total of 5076 potentially relevant records were identified through database searching. After removing duplicates, we obtained a total of 2518 citations for initial screening. Title and abstract screening resulted in 177 papers for detailed review. After examination of full-text articles, five eligible studies were finally included in the meta-analysis [33,41–44] (Fig 1) (Detailed parameter values for the five included clinical trials are provided in S2 Table).

For the hospital survey portion of the study, 1 secondary and 1 tertiary hospital in eastern China were selected. We collected direct medical cost data for diabetes-related complications incurred between 2010 and 2014 in both hospitals, which included diagnosis, medications, medical materials, operations, nursing, and other expenses, and synthesized these data to form an alternative cost profile. These variables were evaluated in the sensitivity analysis.

## Model Inputs

### Patient profile and treatment strategy

This study included Chinese patients with T2DM who failed to achieve adequate glucose control following MET monotherapy and required add-on treatments. Demographic and risk factor profiles were primarily synthesized from meta-analysis of the five included head-to-head studies [33,41–44]. When information pertaining to a specific variable, such as patient height and proportion of patients who smoked, was not available, data from large national observational survey studies were used as a reference [45–46] (Table 1).

**Table 1:** Demographic and Risk Factors.

Variable ^a^	Mean or percentage	Standard Error
**Baseline demographics**		
Age, year	54.62	3.86
Female, value: 0–1	0.45	0.02
Duration of diabetes, year	1.16	0.12
Height, meter	1.64	0
Current smokers, value: 0–1	0.183	0.0046
**Modifiable risk factors**		
HbA1c, %	7.89	0.09
Total-cholesterol, mmol/L	4.98	0.10
HDL cholesterol, mmol/L	1.11	0.06
SBP, mmHg	120.23	4.17
Weight, kg	70.76	2.92

HbA1c, glycated hemoglobin; HDL, high-density lipoprotein; SBP, systolic blood pressure.

^**a**^ Most variables were obtained from five head-to-head studies [33,41–44]; those not available (height and current smokers) were obtained from published studies [45–46].

Patients with T2DM started with SAXA+MET (treatment arm) or ACAR+MET (control arm), and when patients in either arm failed to reach target HbA1c level, therapy escalation for insulin therapy would take place. In accordance with 2013 clinical guidelines from the Chinese Diabetes Society, the HbA1c threshold values for a switch in medication were defined as 7.0% [5]. An alternative HbA1c threshold value of 7.5% was investigated in a univariate sensitivity analysis.

### Clinical and adverse event data

The clinical effects evaluated included treatment-induced impacts on HbA1c, body weight, SBP and cholesterol; and rates of adverse events, including hypoglycemia and GI adverse events, were also evaluated for each arm. These data were obtained from meta-analysis of the five head-to-head studies [33,41–44]. Hypoglycemia is differentiated as symptomatic and severe ones in the model, but all the head-to-head studies did not clearly differentiate between symptomatic and severe hypoglycemia episodes. Therefore, we estimated that a rate of 2.18% represented the proportion of severe cases out of all hypoglycemia events [40]. The efficacy of insulin therapy used the inherent therapy profile of the Cardiff model [47] (Table 2). Regardless of the treatment effect on HbA1c, its value was assumed to increase progressively and gradually. The natural progression of weight gain (0.1 kg/y) was used for both treatment and control arms in the model.

**Table 2:** Clinical Input Variables.

	SAXA+MET ^a^		ACAR+MET ^a^		Insulin ^c^
Variable	Mean	SE	Mean	SE	Mean
HbA1c change, %	-1.02	0.11	-0.81	0.07	−1.11
Weight change, kg	-1.88	0.74	-0.26	0.76	1.9
SBP change, mmHg	-1.79	1.22	-1.83	1.11	0
Total-cholesterol change, mmol/l	-0.23	0.22	-0.13	0.12	0
HDL cholesterol change, mmol/l	0.06	0.05	0.01	0.05	0
Probability symptomatic hypoglycemia	0.018	0.009 ^b^	0.009	0.0064 ^b^	0.616
Probability severe hypoglycemia	0.0004	0.0013 ^b^	0.0002	0.0009 ^b^	0.022
Probability gastrointestinal adverse events	0	0 ^b^	0.1	0.02 ^b^	0

ACAR, acarbose; HbA1c, glycated hemoglobin; HDL, high-density lipoprotein; MET, metformin; SAXA, saxagliptin; SBP, systolic blood pressure; SE, standard error.

^**a**^ Variables were taken from five head-to-head studies [33,41–44];

^**b**^ Calculated as √ rate (1–rate)/numbers of subjects.

^**c**^ Efficacy of insulin used the inherent therapy profile of Cardiff model, in which all SE are 0 [47].

### Costs

Input costs were those related to drug acquisitions, diabetes-related complications, adverse events, and body weight changes. From the payer’s perspective, only country-specific direct medical costs were considered in this study, and all costs were inflated to 2014 values (Chinese yuan) using the Chinese Consumer Price Index [48].

Annual treatment costs of target drugs were calculated using the highest retail price from the most recent official drug price list [49–50] and daily drug dosages from the five head-to-head studies [33,41–44]. The annual cost of MET was estimated from Hou et al [51]. Insulin cost per kilogram weight per day was assumed to be ¥0.137 based on the inherent therapy profile of the Cardiff model (Table 3).

**Table 3:** Annual Treatment Costs (2014 Chinese yuan).

Drug (Brand)	Specification	Highest Retail Price, ¥	Daily Dose, mg/d	Annual Treatment Cost, ¥	Annual Metformin Cost, ¥	Total Cost, ¥
Saxagliptin (Onglyza)	5mg x7 tablets	69.65	5	3631.75 ^a^	1577.28 ^c^	5209.03
Acarbose (Glucobay)	50mg x30 tablets	74.20	150	2708.30 ^b^	1577.28 ^c^	4285.58

^**a**^ Official drug price for saxagliptin in eastern China according to Chinese Price Bureau [49].

^**b**^ Official drug price for acarbose in eastern China according to Chinese Price Bureau [50].

^**c**^ Obtained from Hou et al. [51]. The cost for metformin is ¥366.9 per 12 weeks, and thus the cost of metformin = 366.9x 4 = 1467.6. Convert to 2014 yuan using the Chinese Consumer Price Index from 2013 to 2014, annual cost of metformin = 1577.28.

Costs associated with diabetes-related complications were split into fatal or nonfatal costs, which were applied in the cycle in which the event occurred. For those surviving the event, maintenance costs were applied in all subsequent years until patient death or the simulation ends. The costs were estimated based largely on Gao et al [52]; when the direct cost of a diabetes-related complication (eg, ulcer) was unavailable, we used data from the hospital survey and other published studies [53–54] (Table 4).

**Table 4:** Annual Direct Medical Costs for Diabetes-Related Complications (2014 Chinese Yuan).

	Fatal		Nonfatal		Maintenance	
Event ^a^	Mean	SE	Mean	SE	Mean	SE
Ischemic heart disease	--	0	39,041.39	0	6969.85	0
Myocardial infarction	46,547.02	0	46,547.02	0	10,692.45	0
Congestive heart failure	15,479.64	0	15,479.64	0	9409.36	0
Stroke	14,059.41	0	18,141.13	0	8169.26	0
Blindness	--	--	12,047.60	0	9297.78	0
End-stage renal disease	--	--	114,640.49	0	91,981.79	0
Amputation	18,232.95	0	18,232.95	0	14,533.60	0
Ulcer	0	0	13,989.07	443.2	4923.52	0

^**a**^ Most variables are taken from Gao et al. [52]. Costs of ulcer were obtained from hospital survey and other published studies [53–54].

The treatment cost of severe hypoglycemia (¥3829.96) was abstracted from Zheng et al, which investigated direct medical costs for episodes of hypoglycemia in China [40]; treatment costs of GI adverse events were assumed to be 0 because published evidence of the costs of these adverse events were not available, and GI effects usually do not need to be treated with medicines. Meanwhile, we assumed two different annual costs of ¥200 and ¥1000 for GI events as alternative scenarios in the univariate sensitivity analyses based on the interview of physicians in the hospital. Body mass index (BMI)-related prescription costs which are relate to increased prescribing costs per BMI unit, were calculated and estimated from a follow-up observational study in China [55] (Table 5).

**Table 5:** BMI-Related Prescription Costs (2014 Chinese yuan) ^a^^,^ ^b^.

BMI	Annual Cost	BMI	Annual Cost	BMI	Annual Cost
20	0	27	8189	34	23,751.2
21	0	28	10,412.2	35	25,974.4
22	0	29	12,635.4	36	28,197.6
23	0	30	14,858.6	37	30,420.8
24	1519.5	31	17,081.7	38	32,643.9
25	3742.7	32	19,304.9	39	34,867.1
26	5965.9	33	21,528.1	40+	37,090.3

BMI, body mass index.

^**a**^ Obtained from Guo et al.[55], and BMI-related prescription costs are relate to increased prescribing costs per BMI unit.

^**b**^ It was assumed that the starting point BMI = 25, cost per month = ¥246.8, and the slope (cost per month/BMI) = ¥146.6 in 2007. For BMI ≤23, the cost was set to 0.

### Utilities

Because there were no country-specific utility decrements for diabetes-related events in China, we mainly adopted data from the UKPDS 62 study [56], excluding end-stage renal disease (ESRD) and blindness [57], BMI-related changes [58], hypoglycemia episodes [59], and GI adverse events [60] which were obtained from other studies (Table 6).

**Table 6:** Utility Decrements.

	Utility Decrement	
Event Disutilities ^a^	Year 1	Subsequent Year
Ischemic heart disease	0.090	0.090
Myocardial infarction	0.055	0.055
Congestive heart failure	0.108	0.108
Stroke	0.164	0.164
Blindness	0.074	0.074
End-stage renal disease	0.263	0.263
Amputation	0.280	0.280
Ulcer	0.059	0.059
Symptomatic hypoglycemia	0.0142	0.000
Severe hypoglycemia	0.047	0.000
Gastrointestinal adverse events	0.04	0.000
BMI-related changes		
Per unit decrease in BMI	0.0171	0.0171
Per unit increase in BMI	0.0472	0.0472

BMI, body mass index.

^**a**^ Most variables are taken from the UKPDS 62 study [56]; end-stage renal disease and blindness [57], BMI-related changes [58], hypoglycemia [59], and GI adverse events [60] were obtained from other studies.

### Sensitivity analyses

The impacts of uncertainty and variability around the model inputs were tested by both a series of univariate sensitivity analyses and a probabilistic sensitivity analysis (PSA). Various assumptions about parameters, including baseline demographics, costs and utility decrements associated with diabetes-related complications, body weight changes and other variables, were assessed in univariate sensitivity analyses. All sensitivity analyses were conducted for 1000 patients over 40 years, and a scatter plot of the incremental cost-effectiveness ratios (ICERs) and a cost-effectiveness acceptability curve (CEAC) were generated in the PSA.

## Results

### Predicted health events and costs

**Fig 2:**
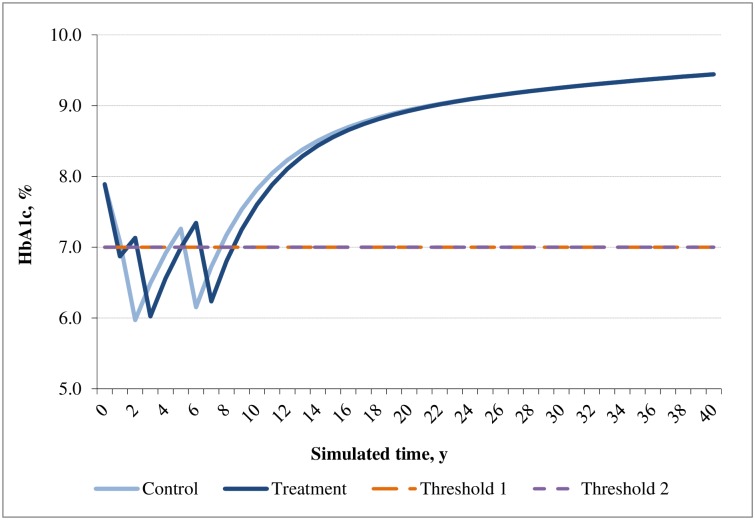
Simulated progression of HbA1c in the treatment (saxagliptin+metformin) and control (acarbose+metformin) arms over the modeled time horizon.

**Fig 3:**
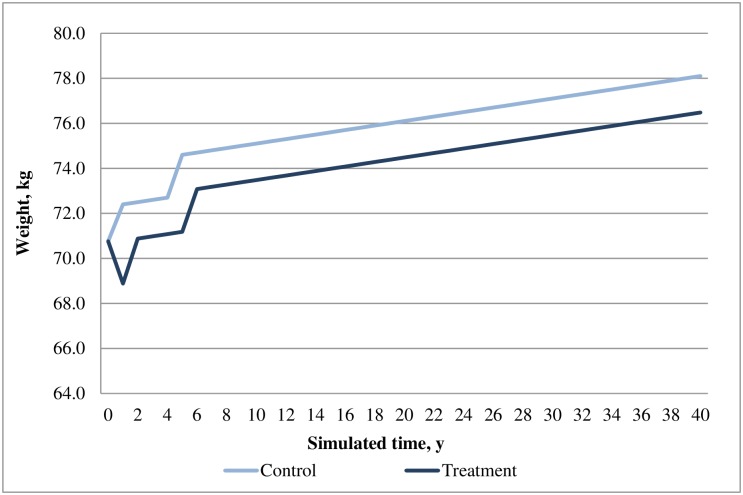
Simulated progression of body weight in the treatment (saxagliptin+metformin) and control (acarbose+metformin) arms over the modeled time horizon.

**Fig 4:**
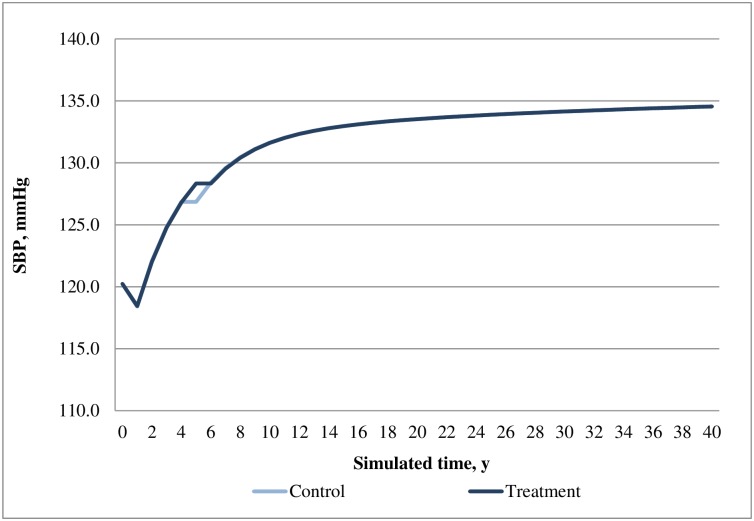
Simulated progression of SBP in the treatment (saxagliptin+metformin) and control (acarbose+metformin) arms over the modeled time horizon.

**Fig 5:**
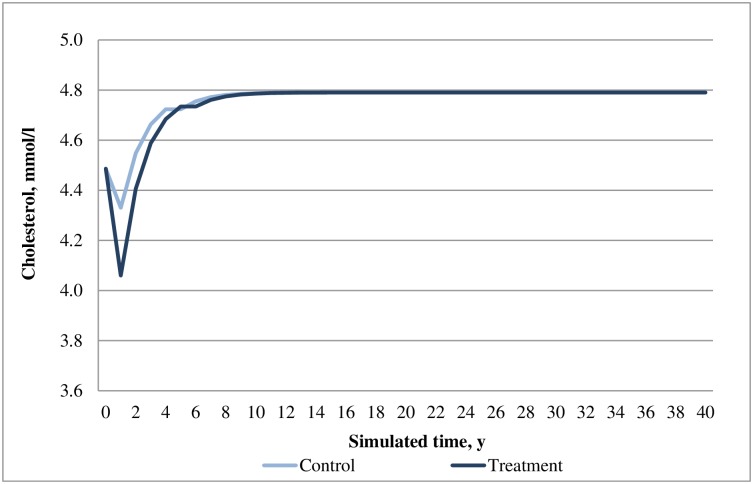
Simulated progression of cholesterol in the treatment (saxagliptin+metformin) and control (acarbose+metformin) arms over the modeled time horizon.

In the base case analysis, the SAXA+MET cohort predicted lower incidences of most cardiovascular events, hypoglycemia events and fatal events as compared with that of the ACAR+MET cohort. Consistent with the differences in cases of diabetes-related events, the costs for most of these events were lower in SAXA+MET than that of ACAR+MET, except for congestive heart failure, stroke and nephropathy which were lower in ACAR+MET. Although drug treatment costs were higher in SAXA+MET, this disadvantage was offset by its much lower BMI-related prescription costs and hypoglycemia costs as compared with ACAR+MET. Overall, SAXA+MET was associated with lower total costs than that of ACAR+MET (Table 7). The time courses of HbA1c, weight, SBP and cholesterol profiles are presented in Figs 2–5.

**Table 7:** Base Case Results for Saxagliptin plus Metformin Compared with Acarbose plus Metformin (2014 Chinese yuan).

Total Events Predicted	ACAR+MET		SAXA+MET		Difference	Total Costs, ¥	ACAR+MET	SAXA+MET
Macrovascular	Non-Fatal	Fatal	Non-Fatal	Fatal		Macrovascular		
Ischaemic Heart Disease	118.58	0	117.93	0	−0.65	Ischaemic Heart Disease	8,206,814	8,140,526
Myocardial Infarction	131.90	170.12	131.06	168.87	−2.08	Myocardial Infarction	17,241,229	17,098,942
Congestive heart Failure	67.48	7.41	67.57	7.39	0.07	Congestive heart Failure	3,373,087	3,382,683
Stroke	66.21	19.00	66.39	18.96	0.15	Stroke	3,661,361	3,672,436
Microvascular	Non-Fatal	Fatal	Non-Fatal	Fatal		Microvascular		
Blindness	70.19	0	69.83	0	−0.36	Blindness	4,511,495	4,508,078
Nephropathy	17.13	1.93	17.32	1.92	0.17	Nephropathy	6,023,505	6,177,940
Amputation	27.69	3.13	27.27	3.07	−0.48	Amputation	1,752,428	1,722,139
Fatal						Hypoglycemia	1,229,401	1,152,822
Macrovascular		196.52		195.21	−1.31	Treatment ^a^	58,718,868	60,074,731
Microvascular		5.06		4.99	−0.07	BMI Costs	130,768,244	110,820,076
						Total	235,486,432	216,750,373
Cost-Effectiveness (per patient)	ACAR+MET		SAXA+MET		Difference	Hypoglycemia ^b^	ACAR+MET	SAXA+MET
Discounted Cost	235486.43		216750.37		−18,736	Symptomatic	12589	12025
Discounted QALYs	12.361		12.845		0.48	Severe	449	429
Discounted Life Years	15.587		15.608		0.02			
Cost per QALY				Dominates	−38,640			
Cost per Life Year				Dominates	−918,030			

ACAR, acarbose; BMI, body mass index; LY, life-year; MET, metformin; QALY, quality-adjusted life-year; SAXA, saxagliptin.

^a^ Treatment cost included cost of insulin and cost of rescue therapy with insulin. Analysis based on 1000 patients.

^**b**^ Hypoglycemia in both the treatment and the control group included hypoglycemic events generated by insulin and rescue therapy.

### Incremental cost-effectiveness ratio

For an individual patient, the total discounted costs accumulated over the lifetime on SAXA+MET was ¥18,736 lower than ACAR+MET; but the QALYs gained with SAXA+MET was 0.48 more than ACAR+MET. This resulted in a cost saving of ¥38,640 per QALY gained with SAXA+MET (i.e., ICER was −¥38,640/QALY gained for SAXA+MET versus ACAR+MET), which indicated that SAXA+MET would lead to better utility and decreased costs for patients (Table 7).

### Parameters influencing the incremental cost-effectiveness ratio

**Fig 6:**
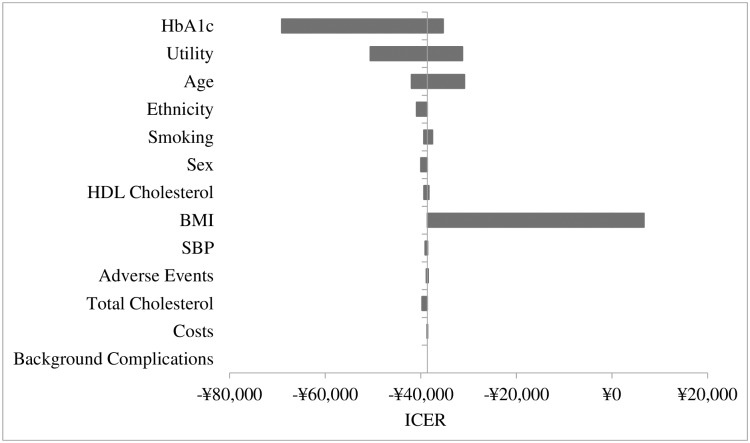
Tornado diagram of the univariate sensitivity analysis.

In the sensitivity analyses, an initial tornado model, run to explore factors with the greatest influence on the cost-effectiveness results, suggested the primary importance of BMI, HbA1c and utility (Fig 6). Subsequently, detailed univariate sensitivity analyses were carried out on several key parameters.

When the baseline HbA1c was decreased by 20%, the reported ICER was −¥67,630/QALY, showing that SAXA+MET still dominant over ACAR+MET. Alternative HbA1c threshold value for therapy switch was also investigated, reporting an ICER of −¥60,662/QALY. Further, utility decrement and cost associated with per unit BMI gain were varied. In the scenario in which the utility decrement per unit BMI gain halved, the incremental QALYs decreased from 0.48 to 0.27, but SAXA+MET kept dominant over ACAR+MET with a reported ICER of −¥69,725/QALY. In the scenario in which an alternative utility decrement per unit BMI change (i.e., the absolute value of the weight utility related to the gain or loss per unit BMI was 0.014) was used, SAXA+MET was still cost-effective compared with ACAR+MET, reporting an ICER of −¥105,897/QALY which was 174% higher than that of base case (−¥38,640/QALY). As expected, a decrease in BMI-related prescription costs would have a negative effect on ICER. When the BMI-related prescription cost was halved, the incremental cost saving decreased from ¥18,736 to ¥8,762, with an ICER of −¥18,070/QALY. Only when the BMI-related prescription cost was excluded from the model, the converse occurred: SAXA+MET cost ¥1,212 more than ACAR+MET with an ICER of ¥2,500/QALY, within the acceptable range of 2014 GDP per capita of China (¥46,629) [61]. (Table 8).

**Table 8:** Sensitivity Analyses for Saxagliptin plus Metformin versus Acarbose plus Metformin, Results per Patient (2014 Chinese yuan).

Sensitivity Analysis*	Difference in Cost, ¥	Difference in QALY	ICER, ¥
**Univariate sensitivity analysis**			
Baseline HbA1c was decreased by 20%	−26,006	0.38	−67,630
HbA1c threshold value for insulin therapy and rescue therapy 7.5%	−24,367	0.40	−60,662
Utility decrement per unit BMI gain halved	−18,736	0.27	−69,725
Utility weight 0.014 per unit BMI decrease and −0.014 per unit BMI increase	−18,736	0.18	−105,897
BMI-related prescription costs halved	−8,762	0.48	−18,070
BMI-related prescription costs set to be 0	1,212	0.48	2,500
SAXA annual therapy cost equal to ACAR	−20,550	0.48	−42,381
SAXA annual therapy cost halved	−22,303	0.48	−45,995
ACAR annual therapy cost doubled	−21,441	0.48	−44,219
Cost of GI adverse events set to be ¥200	−18,756	0.48	−38,681
Cost of GI adverse events set to be ¥1000	−18,836	0.48	−38,847
Cost of severe hypoglycemia doubled	−18,813	0.48	−38,798
GI adverse events in ACAR+MET doubled	−18,736	0.49	−38,330
Utility decrement of GI adverse events doubled	−18,736	0.49	−38,329
Probability of hypoglycemia of SAXA+MET equal to ACAR+MET	−18,737	0.49	−38,622
Utility decrement of hypoglycemia doubled	−18,736	0.49	−37,961
Discount rate (costs and benefits) 3.5%	−17,647	0.46	−38,180
Alternative diabetes-related complications costs	−18,729	0.48	−38,626
**Probabilistic sensitivity analysis**	−21,999	0.47	−46,815

ACAR, acarbose; BMI, body mass index; GI, gastrointestinal; HbA1c, glycated hemoglobin; ICER, incremental cost-effectiveness ratio; MET, metformin; QALY, quality-adjusted life-year; SAXA, saxagliptin.

*Analysis based on 1000 patients. Everything else is as described for the base case analysis.

Drug treatment costs played an important role in the total costs of both arms in the base case analysis and further influenced ICER result. Scenarios analyses demonstrated that either by adjusting the annual treatment cost of SAXA or ACAR (setting the cost of SAXA to be equal to that of ACAR, halving the cost of SAXA, or doubling the cost of ACAR), the cost saving increased from ¥38,640/QALY to ¥42,381/QALY, ¥45,995/QALY or ¥44,219/QALY gained with SAXA+MET, respectively; SAXA+MET gained more dominance over ACAR+MET as compared with that of the base case. GI adverse events and hypoglycemia were commonly observed in the treatment of T2DM, which might have an effect on both cost and utility. Alternative treatment costs of GI events or hypoglycemia in the sensitivity analyses, resulting in a little changes in cost saving gained by SAXA+MET compared to that of base case. When GI adverse events in ACAR+MET doubled or hypoglycemia of SAXA+MET equal to ACAR+MET, the incremental QALYs gained by SAXA+MET increased from 0.48 to 0.49. Alternative annual discount rate for costs and benefits and alternative costs of diabetes-related complications also had some influences on the magnitude of the cost-effectiveness results but did not change the results; SAXA+MET kept dominant over ACAR+MET with better utility and lower cost (Table 8).

**Fig 7:**
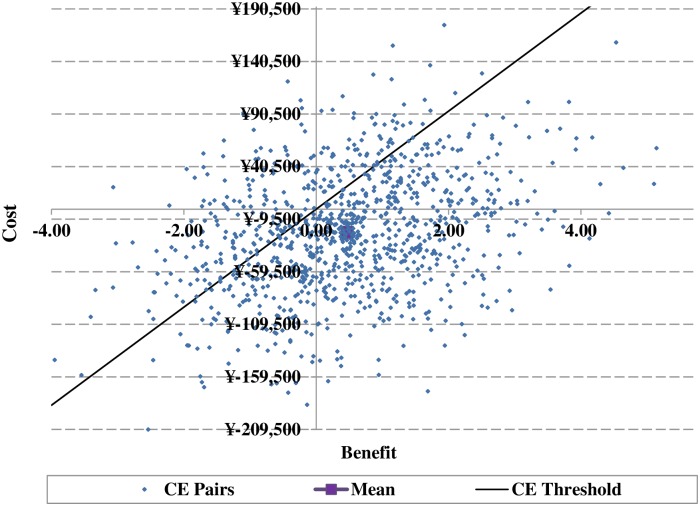
Scatter plot of incremental cost-effectiveness ratios for the treatment (saxagliptin+metformin) arm versus control (acarbose+metformin) arm with a CE threshold value of ¥46,629 (GDP per capita in China in 2014).

**Fig 8:**
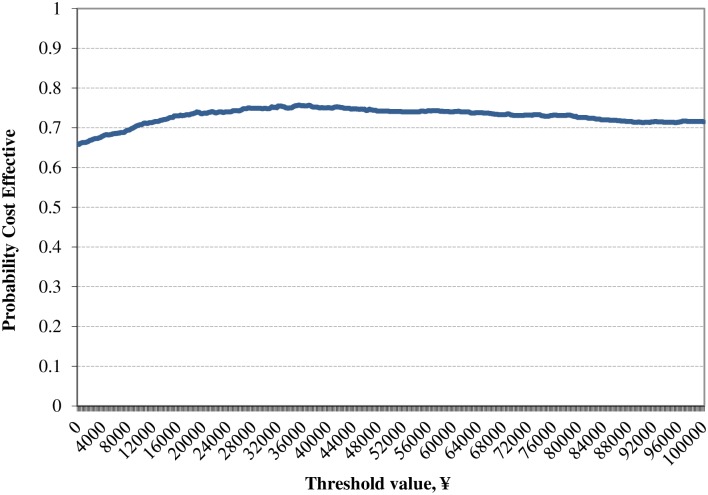
Cost effectiveness acceptability curve for the treatment (saxagliptin+metformin) arm versus control (acarbose+metfromin) arm.

In the PSA, the incremental QALYs gained for SAXA+MET versus ACAR+MET was lower than that of the base case analysis (0.47 versus 0.48); but the incremental cost saving was higher than that of the base case (¥21,999 versus ¥18,736). Thus reporting a cost saving of ¥46,815/QALY gained for SAXA+MET versus ACAR+MET, higher than that of the base case. Fig 7 shows the ICER scatter plot based on the PSA; the points were distributed across all 4 quadrants, with 74.7% of points lying in the southeast quadrants, suggesting cost-effective of SAXA+MET compared with ACAR+MET. Fig 8 shows the CEAC for the base case analysis based on the PSA.

## Discussion

This is the first economic evaluation study using the Cardiff Diabetes Model to determine the long-term economic and health impact of SAXA versus ACAR as add-on therapy to MET for Chinese patients with T2DM who were inadequately controlled following MET monotherapy. The results indicated that the combination therapy of SAXA+MET was dominant over ACAR+MET, with a little QALYs gain and lower costs. The results remained consistent under a series of assumptions in the sensitivity analyses.

Because there is a lack of published studies estimating either the cost or benefit of SAXA compared with ACAR in treating T2DM, we reviewed available literature on the topic of DPP-4i drugs versus ACAR, regardless of the ongoing dearth of cost-effectiveness studies. A 24-week, multi-center, double-blind, randomized trial comparing the DPP-4i vildagliptin (100mg/d) with ACAR (up to 300mg/d) monotherapy in patients with T2DM demonstrated that vildagliptin had similar glycemic efficacy to ACAR but with fewer GI adverse events and better tolerability [62]. Because SAXA (5mg/d) was proved to have almost similar glycaemic control and incidence of adverse events to vildagliptin (100mg/d) as add-on therapy in Chinese patients [63], it can be inferred that SAXA may have non-inferiority efficacy and better safe profile compared with ACAR (300mg/d). In this condition, the disadvantage of SAXA in drug cost might no longer exist in our study, as the drug cost of ACAR doubled compared with that in the base case (for the dose of ACAR was 150mg/d in the base case analysis). Scenario analysis on doubling costs of ACAR confirmed that SAXA+MET gained more dominance over ACAR+MET than that of base case. In addition, systematic reviews and meta-analyses indicate that DPP-4i drugs in comparison with AGIs achieved similar benefit profiles (modest glucose control and neutral effects on weight) and were well tolerated, with lower risk of hypoglycemia and other adverse events, whereas AGIs were associated with frequent GI adverse events and a frequent dosing schedule [6,64–66]. Chinese T2DM Clinical Guidelines recommend both DPP-4is and AGIs as second-line therapies in treating patients with T2DM, whereas AGIs are excluded from the American Diabetes Association guidelines [4–5].

A patient-centered treatment approach for T2DM should take into account the individual patient’s characteristics, preferences and values and balance the need to optimize efficacy with the need to minimize adverse events [6]. In this study, sensitivity analyses on GI adverse events and hypoglycemia had demonstrated the influence of adverse events on the incremental QALYs and cost savings. Our study highlighted the fact that efficacy and tolerability are no longer the only criteria used in evaluating T2DM treatments; there is also an urgent need to minimize adverse events. Adverse events, such as hypoglycemia, weight gain and GI symptoms, may interfere with the attainment of stringent blood glucose control, either through suboptimal dosing and/or poor medication adherence, and increase the risk of cardiovascular complications, further impacting the patient’s QOL [7,9–10,67].

Medication nonadherence or suboptimal adherence is one of the leading public health challenges, particularly in the case of medications for chronic diseases like diabetes [68–69]. Nonadherence or suboptimal adherence to a T2DM regimen is associated with poor blood glucose control, increased risk of complications and mortality, and higher healthcare resource utilization and costs for patients [8,12–15]. Therefore, improving patient compliance to medication is crucial in T2DM management. Medication adherence may be negatively impacted by a number of factors, including adverse events, frequent dosing schedule, inconvenient administration, lack of knowledge about diabetes and high out-of-pocket expenses [8,20–21,70]. Head-to-head studies highlighted poorer patient compliance in ACAR+MET than that in SAXA+MET (rates of misuse or missing use of drugs: 19.1%-20% versus 5.6%-6.4%), which was partly attributed to the common GI adverse events, frequent dosing schedule (3 times/d) and inconvenient administration of ACAR [27,33,41]. In comparison with ACAR, the SAXA dosage of 1 tablet per day taken orally is easier to administer, particularly for elderly patients with memory loss, potentially leading to greater adherence [21,32–33]. Drugs with proven efficacy, favorable adverse event profiles, and easy administration like SAXA are needed to enhance patient QOL.

With the growth of national economies and concomitant increase in income levels, people are more focused on QOL and may be willing to spend more money for convenient drug administration and reduced drug adverse effects. Although the use of SAXA is somewhat limited by its slightly high cost [4,6], our study showed that SAXA was a cost-effective treatment alternative for patients with T2DM compared with ACAR, with a favorable adverse event profile, ease of administration, and improved long-term health outcomes as well as lower costs.

SAXA is a well-tolerated drug that effectively controls blood glucose levels and has a low risk of hypoglycemia, weight gain, and GI adverse events, making it possible to increase patient QOL and allow for better medication adherence [21,28–31]. Given the poor management, high rate of diabetes-related deaths, and suboptimal medication adherence of diabetes patients in China, SAXA may be a beneficial drug for second-line therapy in this patient population, particularly for those who are bothered by the treatment-induced adverse events and inconvenient administration of other antidiabetic drugs.

There were several limitations to our study. First, there is a paucity of long-term follow-up data from well-designed clinical or epidemiologic studies directly comparing the treatment effects of SAXA+MET versus ACAR+MET in patients with T2DM in China; only five short-term head-to-head trials on this topic was identified. We therefore used the aforementioned trials to project long-term outcomes of both treatments using UKPDS 68 equations, which might cause uncertainty in the input parameters and bias to real-world settings. Second, only direct medical costs were investigated in our study, which neglected the considerable indirect costs of diabetes-related events (hypoglycemia, weight gain, GI adverse events) on productivity. Moreover, total costs in the ACAR+MET cohort were underestimated because of the unavailability of expenses related to GI adverse events, which may have undermined the comparability of the treatment arms to some extent. Third, because there were no country-specific utilities for diabetes-related complications and adverse events in China, utilities from the UKPDS 62 and other published studies of foreign populations were used, which might introduce a bias.

## Conclusion

This study showed from a payer’s perspective that SAXA+MET is a cost-effective treatment alternative compared with ACAR+MET for patients with T2DM inadequately controlled on MET monotherapy in China, with a little QALYs gain and lower costs. SAXA is an effective, well-tolerated drug with a low incidence of adverse events and ease of administration. It is anticipated to favor patients who wish to avoid the treatment-induced adverse events and inconvenient administration of other antidiabetic drugs, with the goal of improving patient with effective second-line therapy for T2DM treatment.

## Supporting Information

S1 Appendix Selection Criteria. (PDF)

S1 Checklist PRISMA 2009 Checklist. (DOC)

S1 Table Search Strategy. (PDF)

S2 Table Detailed Parameter Values for the Five Included Clinical Trials. (PDF)
