# Supplementary material for: Cost-Effectiveness of Saxagliptin versus Acarbose as Second-Line Therapy in Type 2 Diabetes in China
Source: PLoS One. 2016 Nov 22;11(11):e0167190. doi: 10.1371/journal.pone.0167190 (PMC5119856; doi:10.1371/journal.pone.0167190)
Supplement: S1 Appendix — (PDF) [file pone.0167190.s001.pdf]

Selection Criteria

Inclusion criteria:

(1) Only randomized controlled trials were eligible;

(2) Head-to-head studies compared clinical effect of saxagliptin versus acarbose as add-on therapy to metformin;

(3) Studies on Chinese patients with type 2 diabetes (T2DM) who are 18 years or older;

(4) Studies on patients not adequately controlled by metformin; expect for diet or/and exercise, only saxagliptin and metformin in saxagliptin arm, and acarbose and metformin in acarbose arm were allowed;

(5) Studies written in Chinese or English;

(6) Studies had glycosylated hemoglobin (HbA_{1c}) as the primary outcome indicator; and systolic blood pressure (SBP), body weights, cholesterol, or hypoglycemia as outcome indicator;

(7) Studies with a follow-up duration of 12 weeks or longer;

Exclusion criteria:

(1) Studies not related to or not targeted at saxagliptin (or acarbose), including studies merely on dipeptidyl peptidase-4 inhibitor (DPP-4i) or alpha-glucosidase inhibitors;

(2) Studies only provided information about saxagliptin (or acarbose), including introduction of pharmacokinetics, pharmacologic properties, pharmacodynamics, clinical use or mechanism of action;

(3) Studies were monotherapy design;

(4) Studies not targeted at (saxagliptin + metformin) versus (acarbose + metformin);

(5) Studies not targeted at T2DM, including impaired glucose tolerance (IGT), impaired fasting glucose (IFG), gestational diabetes or type 1 diabetes (T1DM);

(6) Studies on cell lines or animals;

(7) Literature reviews or meta-analysis;

(8) Meeting abstracts;

(9) Economic evaluation studies;

(10) Letters or comments;

(11) Duplicate;
