# Supplementary material for: Cost-Effectiveness of Saxagliptin versus Acarbose as Second-Line Therapy in Type 2 Diabetes in China
Source: PLoS One. 2016 Nov 22;11(11):e0167190. doi: 10.1371/journal.pone.0167190 (PMC5119856; doi:10.1371/journal.pone.0167190)
Supplement: S1 Table — (PDF) [file pone.0167190.s003.pdf]

Search Strategy

Database 1: PubMed

Keywords	Query	Items found
Saxagliptin		
#1	Search "saxagliptin" [Supplementary Concept] OR "5-hydroxysaxagliptin" [Supplementary Concept]	224
#2	Search ((("saxagliptin" [Supplementary Concept] OR "5-hydroxysaxagliptin" [Supplementary Concept])) OR saxagliptin) OR Onglyza	474
Acarbose		
#3	Search "Acarbose"[Mesh] OR "acarbose byproduct, component C" [Supplementary Concept] OR "acarbose 7-phosphotransferase" [Supplementary Concept] OR "acarbose 7-phosphate" [Supplementary Concept]	1164
#4	Search ((("Acarbose"[Mesh] OR "acarbose byproduct, component C" [Supplementary Concept] OR "acarbose 7-phosphotransferase" [Supplementary Concept] OR "acarbose 7-phosphate" [Supplementary Concept])) OR acarbose) OR Glucobay	2064
Saxagliptin OR Acarbose: #2 OR #4		
#5	Search ((((("saxagliptin" [Supplementary Concept] OR "5-hydroxysaxagliptin" [Supplementary Concept])) OR saxagliptin) OR Onglyza)) OR (((("Acarbose"[Mesh] OR "acarbose byproduct, component C" [Supplementary Concept] OR "acarbose 7-phosphotransferase" [Supplementary Concept] OR "acarbose 7-phosphate" [Supplementary Concept])) OR acarbose) OR Glucobay)	2533
Type 2 diabetes		
#6		101455

	Search "Diabetes Mellitus, Type 2"[Mesh] OR "Polyendocrinopathies, Autoimmune"[Mesh]	
#7	Search (((("Diabetes Mellitus, Type 2"[Mesh] OR "Polyendocrinopathies, Autoimmune"[Mesh])) OR Type 2 diabetes) OR non-insulin-dependent diabetes mellitus) OR T2DM	145958
Chinese		
#8	Search "China"[Mesh] OR "Taiwan"[Mesh]	151962
#9	Search "Asian Continental Ancestry Group"[Mesh]	54349
#10	Search (((("China"[Mesh] OR "Taiwan"[Mesh])) OR "Asian Continental Ancestry Group"[Mesh]) OR China) OR Chinese	1225406
(Saxagliptin OR Acarbose) AND (Type 2 diabetes) AND Chinese: #5 AND #7 AND #10		
#11	Search (((((((("saxagliptin" [Supplementary Concept] OR "5-hydroxysaxagliptin" [Supplementary Concept])) OR saxagliptin) OR Onglyza)) OR (((("Acarbose"[Mesh] OR "acarbose byproduct, component C" [Supplementary Concept] OR "acarbose 7-phosphotransferase" [Supplementary Concept] OR "acarbose 7-phosphate" [Supplementary Concept])) OR acarbose) OR Glucobay))) AND ((((("Diabetes Mellitus, Type 2"[Mesh] OR "Polyendocrinopathies, Autoimmune"[Mesh])) OR Type 2 diabetes) OR non-insulin-dependent diabetes mellitus) OR T2DM)) AND ((((("China"[Mesh] OR "Taiwan"[Mesh])) OR "Asian Continental Ancestry Group"[Mesh]) OR China) OR Chinese)	111
(Saxagliptin OR Acarbose) AND (Type 2 diabetes) AND Chinese AND (2009-2016): #11 AND (2009-2016)		
#12	Search (((((((((("saxagliptin" [Supplementary Concept] OR "5-	91

	hydroxysaxagliptin" [Supplementary Concept])) OR saxagliptin) OR Onglyza)) OR (((("Acarbose"[Mesh] OR "acarbose byproduct, component C" [Supplementary Concept] OR "acarbose 7-phosphotransferase" [Supplementary Concept] OR "acarbose 7-phosphate" [Supplementary Concept])) OR acarbose) OR Glucobay))) AND ((((("Diabetes Mellitus, Type 2"[Mesh] OR "Polyendocrinopathies, Autoimmune"[Mesh])) OR Type 2 diabetes) OR non-insulin-dependent diabetes mellitus) OR T2DM)) AND ((((("China"[Mesh] OR "Taiwan"[Mesh])) OR "Asian Continental Ancestry Group"[Mesh]) OR China) OR Chinese))) AND ("2009/01/01"[Date - Publication] : "2016/09/30"[Date - Publication])	
(Saxagliptin OR Acarbose) AND (Type 2 diabetes) AND Chinese AND (2009-2016) AND Humans: #12 AND humans		
#13	Search (((((((((("saxagliptin" [Supplementary Concept] OR "5-hydroxysaxagliptin" [Supplementary Concept])) OR saxagliptin) OR Onglyza)) OR (((("Acarbose"[Mesh] OR "acarbose byproduct, component C" [Supplementary Concept] OR "acarbose 7-phosphotransferase" [Supplementary Concept] OR "acarbose 7-phosphate" [Supplementary Concept])) OR acarbose) OR Glucobay))) AND ((((("Diabetes Mellitus, Type 2"[Mesh] OR "Polyendocrinopathies, Autoimmune"[Mesh])) OR Type 2 diabetes) OR non-insulin-dependent diabetes mellitus) OR T2DM)) AND ((((("China"[Mesh] OR "Taiwan"[Mesh])) OR "Asian Continental Ancestry Group"[Mesh]) OR China) OR Chinese))) AND ("2009/01/01"[Date - Publication] : "2016/09/30"[Date - Publication]) Filters: Humans	54

Database 2: Web of Knowledge

Keywords	Query	Items found
**Saxagliptin**		
# 1	**TS=saxagliptin**<br>*Time span= 2009-2016; Retrieval language =automatic*	927
# 2	**TS=Onglyza**<br>*Time span= 2009-2016; Retrieval language =automatic*	35
# 3	**#2 OR #1**<br>*Time span= 2009-2016; Retrieval language =automatic*	930
**Acarbose**		
# 4	**TS=acarbose**<br>*Time span= 2009-2016; Retrieval language =automatic*	1,828
# 5	**TS=Glucobay**<br>*Time span= 2009-2016; Retrieval language =automatic*	16
# 6	**#5 OR #4**<br>*Time span= 2009-2016; Retrieval language =automatic*	1,831
**Saxagliptin OR Acarbose**		
# 7	**#6 OR #3**<br>*Time span= 2009-2016; Retrieval language =automatic*	2,744
**Type 2 diabetes**		
# 8	**TS=Type 2 diabetes**<br>*Time span= 2009-2016; Retrieval language =automatic*	146,150
# 9	**TS=non-insulin-dependent diabetes mellitus**<br>*Time span= 2009-2016; Retrieval language =automatic*	43,447
# 10	**TS=T2DM**<br>*Time span= 2009-2016; Retrieval language =automatic*	15,336

# 11	**#10 OR #9 OR #8**<br>*Time span= 2009-2016; Retrieval language =automatic*	147,395
**Chinese**		
# 12	**TS=Chinese**<br>*Time span= 2009-2016; Retrieval language =automatic*	406,378
# 13	**TS=China**<br>*Time span= 2009-2016; Retrieval language =automatic*	516,416
# 14	**#13 OR #12**<br>*Time span= 2009-2016; Retrieval language =automatic*	825,311
**(Saxagliptin OR Acarbose) AND (Type 2 diabetes) AND Chinese**		
# 15	**#14 AND #11 AND #7**<br>*Time span= 2009-2016; Retrieval language =automatic*	75

Database 3: Cochrane Library

**Keywords**	**Query**	**Items found**
**Saxagliptin**		
#1	saxagliptin<br>*Publication Year from 2009 to 2016, in Trials*	160
#2	Onglyza<br>*Publication Year from 2009 to 2016, in Trials*	6
#3	#2 or #1	160
**Acarbose**		
#4	acarbose<br>*Publication Year from 2009 to 2016, in Trials*	158
#5	Glucobay<br>*Publication Year from 2009 to 2016, in Trials*	3
#6	#5 or #4	158
**Saxagliptin OR Acarbose**		
#7	#6 or #3	312
**Type 2 diabetes**		
#8	Type 2 diabetes<br>*Publication Year from 2009 to 2016, in Trials*	11692
#9	non-insulin-dependent diabetes mellitus<br>*Publication Year from 2009 to 2016, in Trials*	5407
#10	T2DM<br>*Publication Year from 2009 to 2016, in Trials*	2005
#11	#10 or #9 or #8	12176
**Chinese**		
#12	Chinese<br>*Publication Year from 2009 to 2016, in Trials*	14122
#13	China<br>*Publication Year from 2009 to 2016, in Trials*	18690
#14	#13 or #12	23677
**(Saxagliptin OR Acarbose) AND (Type 2 diabetes) AND Chinese**		
#15	#14 and #11 and #7	49

Database 4: ScienceDirect

**Expert search**	**Query**	**Results**
Search Strategy	(title-abstr-key (saxagliptin) OR title-abstr-key (Onglyza) OR title-abstr-key (acarbose) OR title-abstr-key (Glucobay)) AND (title-abstr-key (Type 2 diabetes) OR title-abstr-key (non-insulin-dependent diabetes mellitus) OR title-abstr-key (T2DM)) AND (title-abstr-key (China) OR title-abstr-key (Chinese))	**8**
Refine your search	Journals; Books; All; -All Sciences-	
Years	2009- 2016	

Database 5: China National Knowledge Infrastructure (CNKI)

**Expert Search**	**Query**	**Items found**
Search Strategy	(SU=沙格列汀 OR SU=沙克列汀 OR SU=安立泽 OR SU=阿卡波糖 OR SU=拜糖平 OR SU=拜糖苹 OR SU=拜唐苹) AND (SU=2 型糖尿病 OR SU=Ⅱ型糖尿病)	**1,903**
Publication Date	2009-01-01 to 2016-09-30	

Database 6: the Chongqing VIP (CQVIP)

**Advanced Search**	**Query**	**Items found**
Search Strategy	(U=沙格列汀+U=沙克列汀+U=安立泽+U=阿卡波糖+U=拜糖平+U=拜糖苹+U=拜唐苹)*(U=2 型糖尿病+U=Ⅱ型糖尿病)	**1361**
Publication Date	2009-2016	

Database 7: WanFang Data

**Expert Search**	**Query**	**Items found**
Search Strategy	(主题:(沙格列汀) + 主题:(沙克列汀) + 主题:(安立泽) + 主题:(阿卡波糖) + 主题:(拜糖平) + 主题：(拜糖苹) + 主题:(拜唐苹)) * (主题:(2 型糖尿病) + 主题: (Ⅱ型糖尿病))	**1,626**
Publication Date	2009-2016	
