# Supplementary material for: Cost-Effectiveness of Saxagliptin versus Acarbose as Second-Line Therapy in Type 2 Diabetes in China
Source: PLoS One. 2016 Nov 22;11(11):e0167190. doi: 10.1371/journal.pone.0167190 (PMC5119856; doi:10.1371/journal.pone.0167190)
Supplement: S2 Table — (PDF) [file pone.0167190.s004.pdf]

Detailed Parameter Values for the Five Included Clinical Trials.

Study 1		Lv CF, Yu P, Zhou SJ, Li CJ, Lv L, Chen R, et al. Efficacy and safety of saxagliptin combined metformin in newly diagnosed type 2 diabetes mellitus patients. Chinese Journal of Diabetes Mellitus. 2013; 5: 759-762.			
Drug		SAXA+MET		ACAR+MET	
No. of patients		90		90	
Study Duration		12 week			
Baseline Demographics		Mean	SD	Mean	SD
Age (years)		44	7	44	7
Proportion female (value:0-1)		0.4833		0.4833	
Duration of diabetes (years)		Newly diagnosed		Newly diagnosed	
Height (m)		NR		NR	
Modifiable Risk Factors		Mean	SD	Mean	SD
HbA1c (%)		7.83	0.29	7.81	0.27
Total-cholesterol (mmol/L)		4.9	0.8	4.8	0.8
HDL cholesterol (mmol/L)		1.11	0.31	1.29	0.29
SBP (mmHg)		111	17	110	17
BMI (kg/m2)		30	6	30	4
Weight (kg)		82	23	82	24
Efficacy		Mean	SD	Mean	SD
HbA1c (%)	Pre-treat	7.83	0.29	7.81	0.27
	Post-treat	6.86	0.39	6.99	0.27
	Change	-0.97	0.38	-0.82	0.30
SBP (mmHg)	Pre-treat	111	17	110	17
	Post-treat	NR		NR	
Total-cholesterol (mmol/L)	Pre-treat	4.9	0.8	4.8	0.8
	Post-treat	NR		NR	
HDL cholesterol (mmol/L)	Pre-treat	1.11	0.31	1.29	0.29
	Post-treat	NR		NR	
BMI (kg/m2)	Pre-treat	30	6	30	4
	Post-treat	29	5	29	4
	change	-1.00	6.08	-1.00	4.38
Weight (kg)	Pre-treat	82	23	82	24
	Post-treat	80	22	80	23
	Change	-2.00	24.66	-2.00	25.76
Adverse Events		Mean	SD	Mean	SD
Hypoglycemia	Probability	0.0111		0.0111	
Gastrointestinal discomfort	Probability	0		0.133	

Study 2		Qian D. Efficacy and safety of saxagliptin combined with metformin in the treatment of the patients with newly diagnosed type 2 diabetes. Chinese Journal of Coal Industry Medicine. 2015; 18: 23-26, 27.			
**Drug**		**SAXA+MET**		**ACAR+MET**	
**No. of patients**		**47**		**47**	
**Study Duration**		**12 Week**			
**Baseline Demographics**		**Mean**	**SD**	**Mean**	**SD**
**Age (years)**		**40.8**	**5.9**	**42.6**	**6.3**
**Proportion female (value:0-1)**		**0.34**		**0.38**	
**Duration of diabetes (years)**		**0.3917**	**0.075**	**0.4167**	**0.1**
**Height (m)**		**NR**		**NR**	
**Modifiable Risk Factors**		**Mean**	**SD**	**Mean**	**SD**
**HbA1c (%)**		**7.3**	**0.9**	**7.7**	**1.1**
**Total-cholesterol (mmol/L)**		**4.7**	**0.9**	**4.5**	**0.7**
**HDL cholesterol (mmol/L)**		**NR**		**NR**	
**SBP (mmHg)**		**109.5**	**13.2**	**112.4**	**14**
**BMI (kg/m2)**		**29.8**	**7.4**	**29.6**	**7.3**
**Weight (kg)**		**80.7**	**7.1**	**80.2**	**7**
**Efficacy**		**Mean**	**SD**	**Mean**	**SD**
**HbA1c (%)**	**Pre-treat**	**7.3**	**0.9**	**7.7**	**1.1**
	**Post-treat**	**6.5**	**0.4**	**6.8**	**0.7**
	**Change**	**-0.8**	**0.83**	**-0.9**	**1.04**
**SBP (mmHg)**	**Pre-treat**	**109.5**	**13.2**	**112.4**	**14**
	**Post-treat**	**NR**		**NR**	
**Total-cholesterol (mmol/L)**	**Pre-treat**	**4.7**	**0.9**	**4.5**	**0.7**
	**Post-treat**	**NR**		**NR**	
**HDL cholesterol (mmol/L)**	**Pre-treat**	**NR**		**NR**	
	**Post-treat**	**NR**		**NR**	
**BMI (kg/m2)**	**Pre-treat**	**29.8**	**7.4**	**29.6**	**7.3**
	**Post-treat**	**30.6**	**7.6**	**29**	**7.2**
	**change**	**0.8**	**8.2175**	**-0.6**	**7.9424**
**Weight (kg)**	**Pre-treat**	**80.7**	**7.1**	**80.2**	**7**
	**Post-treat**	**79.3**	**6.9**	**80.3**	**7.2**
	**Change**	**-1.40**	**7.67**	**0.10**	**7.78**
**Adverse Events**		**Mean**	**SD**	**Mean**	**SD**
**Hypoglycemia**	**Probability**	**0.0213**		**0.0213**	
**Gastrointestinal discomfort**	**Probability**	**NR**		**NR**	

Study 3		Wang MM, Lin S, Chen YM, Shu J, Lu HY, Zhang YJ, et al. Saxagliptin is similar in glycaemic variability more effective in metabolic control than acarbose in aged type 2 diabetes inadequately controlled with metformin. Diabetes Res Clin Pract. 2015; 108: e67-e70.			
Drug		SAXA+MET		ACAR+MET	
No. of patients		41		40	
Study Duration		1 Year			
Baseline Demographics		Mean	SD	Mean	SD
Age (years)		64.3	2.6	65.1	3.1
Proportion female (value:0-1)		0.56		0.53	
Duration of diabetes (years)		12.3	4.5	14.2	4.2
Height (m)		NR		NR	
Modifiable Risk Factors		Mean	SD	Mean	SD
HbA1c (%)		8.26	0.49	8.30	0.37
Total-cholesterol (mmol/L)		5.62	1.35	5.53	1.53
HDL cholesterol (mmol/L)		1.13	0.36	1.20	0.38
SBP (mmHg)		NR		NR	
Weight (kg)		65.2	8.8	63.9	9.7
Efficacy		Mean	SD	Mean	SD
HbA1c (%)	Pre-treat	8.26	0.49	8.30	0.37
	Post-treat	7.44	0.36	7.61	0.38
	Change	-0.82	0.48	-0.69	0.41
SBP (mmHg)	Pre-treat	NR		NR	
	Post-treat	NR		NR	
Total-cholesterol (mmol/L)	Pre-treat	5.62	1.35	5.53	1.53
	Post-treat	5.13	1.27	5.35	1.46
	Change	-0.49	1.44	-0.18	1.64
HDL cholesterol (mmol/L)	Pre-treat	1.13	0.36	1.20	0.38
	Post-treat	1.17	0.35	1.19	0.42
	Change	0.04	0.39	-0.01	0.44
Weight (kg)	Pre-treat	65.2	8.8	63.9	9.7
	Post-treat	63.4	5.6	63.1	7.9
	Change	-1.80	8.33	-0.80	9.76
Adverse Events		Mean	SD	Mean	SD
Hypoglycemia	Probability	0.05		0	

**Gastrointestinal discomfort**	**Probability**	**0**		**0.0444**	

**Study 4**		**Qiao Y, Zhao J, He M, Wang X. Effect of metformin combined with saxagliptin on insulin resistance of newly diagnosed patients with type 2 diabetes. Journal of Practical Diabetology. 2016; 12: 48-50.**			
**Drug**		**SAXA+MET**		**ACAR+MET**	
**No. of patients**		**40**		**40**	
**Study Duration**		**12 Week**			
**Baseline Demographics**		**Mean**	**SD**	**Mean**	**SD**
**Age (years)**		**51.4**	**12.1**	**50.6**	**14.2**
**Proportion female (value:0-1)**		**0.45**		**0.45**	
**Duration of diabetes (years)**		**Newly diagnosed**		**Newly diagnosed**	
**Height (m)**		**NR**		**NR**	
**Modifiable Risk Factors**		**Mean**	**SD**	**Mean**	**SD**
**HbA1c (%)**		**8.6**	**1.9**	**8.5**	**2.1**
**Total-cholesterol (mmol/L)**		**5.06**	**0.68**	**5.1**	**0.75**
**HDL cholesterol (mmol/L)**		**0.91**	**0.56**	**0.93**	**0.43**
**SBP (mmHg)**		**128**	**14**	**124**	**16**
**BMI (kg/m2)**		**24.4**	**5.5**	**23.8**	**4.2**
**Weight (kg)** (calculated by: weight=BMI*height^2, height assumed to be 1.64 m)		**65.63**	**14.79**	**64.01**	**11.30**
**Efficacy**		**Mean**	**SD**	**Mean**	**SD**
**HbA1c (%)**	**Pre-treat**	**8.6**	**1.9**	**8.5**	**2.1**
	**Post-treat**	**6.7**	**2**	**6.9**	**2.3**
	**Change**	**-1.9**	**2.14**	**-1.6**	**2.42**
**SBP (mmHg)**	**Pre-treat**	**128**	**14**	**124**	**16**
	**Post-treat**	**123**	**16**	**122**	**15**
	**Change**	**-5**	**16.52**	**-2**	**17.00**
**Total-cholesterol (mmol/L)**	**Pre-treat**	**5.06**	**0.68**	**5.1**	**0.75**
	**Post-treat**	**5.01**	**0.66**	**4.98**	**0.78**
	**Change**	**-0.05**	**0.73**	**-0.12**	**0.84**
**HDL cholesterol (mmol/L)**	**Pre-treat**	**0.91**	**0.56**	**0.93**	**0.43**
	**Post-treat**	**1.02**	**0.61**	**0.98**	**0.56**
	**Change**	**0.11**	**0.64**	**0.05**	**0.55**
**BMI (kg/m2)**	**Pre-treat**	**24.4**	**5.5**	**23.8**	**4.2**

	**Post-treat**	**22**	**6.3**	**23.4**	**5.8**
	**change**	**-2.4**	**6.50**	**-0.4**	**5.64**
**Weight (kg)** (calculated by: weight=BMI*height^2, height assumed to be 1.64 m)	**Pre-treat**	**65.63**	**14.79**	**64.01**	**11.30**
	**Post-treat**	**59.17**	**16.94**	**62.94**	**15.60**
	**Change**	**-6.46**	**17.48**	**-1.08**	**15.17**
**Adverse Events**		**Mean**	**SD**	**Mean**	**SD**
**Hypoglycemia**	**Probability**	**0**		**0**	
**Gastrointestinal discomfort**	**Probability**	**0**		**0.075**	

**Study 5**		**Wang W, Chen Y, Qian T, Yao W. Effect of saxagliptin combined with metformin on blood glucose, blood pressure and glycosylated hemoglobin of aged patients with type 2 diabetes. Chinese Journal of Gerontology. 2016; 36: 332-333.**			
**Drug**		**SAXA+MET**		**ACAR+MET**	
**No. of patients**		**40**		**40**	
**Study Duration**		**12 Week**			
**Baseline Demographics**		**Mean**	**SD**	**Mean**	**SD**
**Age (years)**		**71.1**	**6.1**	**72.1**	**4.6**
**Proportion female (value:0-1)**		**0.4**		**0.425**	
**Duration of diabetes (years)**		**2.3**	**1.2**	**2.5**	**2.1**
**Height (m)**		**NR**		**NR**	
**Modifiable Risk Factors**		**Mean**	**SD**	**Mean**	**SD**
**HbA1c (%)**		**7.2**	**1.22**	**7.6**	**3.25**
**Total-cholesterol (mmol/L)**		**NR**		**NR**	
**HDL cholesterol (mmol/L)**		**NR**		**NR**	
**SBP (mmHg)**		**132.2**	**8.21**	**134.5**	**7.12**
**BMI (kg/m2)**		**23.5**	**4.65**	**22.7**	**3.86**
**Weight (kg)** (calculated by: weight=BMI*height^2, height assumed to be 1.64 m)		**63.21**	**12.51**	**61.05**	**10.38**
**Efficacy**		**Mean**	**SD**	**Mean**	**SD**
**HbA1c (%)**	**Pre-treat**	**7.2**	**1.22**	**7.6**	**3.25**
	**Post-treat**	**5.4**	**2.44**	**6.9**	**3.53**
	**Change**	**-1.8**	**2.25**	**-0.7**	**3.7209**
**SBP (mmHg)**	**Pre-treat**	**132.2**	**8.21**	**134.5**	**7.12**
	**Post-treat**	**131.3**	**7.65**	**132.7**	**6.98**
	**Change**	**-0.9**	**8.70**	**-1.8**	**7.7238**

**Total-cholesterol (mmol/L)**	**Pre-treat**	**NR**		**NR**	
	**Post-treat**	**NR**		**NR**	
**HDL cholesterol (mmol/L)**	**Pre-treat**	**NR**		**NR**	
	**Post-treat**	**NR**		**NR**	
**BMI (kg/m2)**	**Pre-treat**	**23.5**	**4.65**	**22.7**	**3.86**
	**Post-treat**	**23.2**	**5.57**	**23.1**	**4.76**
	**change**	**-0.3**	**5.65**	**0.4**	**4.78**
**Weight (kg)** (calculated by: weight=BMI*height^2, height assumed to be 1.64 m)	**Pre-treat**	**63.21**	**12.51**	**61.05**	**10.38**
	**Post-treat**	**62.40**	**14.98**	**62.13**	**12.80**
	**Change**	**-0.81**	**15.20**	**1.08**	**12.86**
**Adverse Events**		**Mean**	**SD**	**Mean**	**SD**
**Hypoglycemia**	**Probability**	**NR**		**NR**	
**Gastrointestinal discomfort**	**Probability**	**NR**		**NR**	

ACAR, acarbose; BMI, body mass index; HbA1c, glycated hemoglobin; HDL, high-density lipoprotein; MET, metformin; NR, not reported; SAXA, saxagliptin; SBP, systolic blood pressure.

[a] Mean height of the cohort in the model assumed to be 1.64 m, which is obtained from National Physique Monitoring Bulletin (2014).

[b] For studies 4 and 5, which only provided BMI, weight was calculated by: weight=BMI*height^2, and height was assumed to be 1.64 m.

[c] Mean treatment effect on HbA1c/weight/SBP/TC= Mean $_{post\text{-}treat}$ - Mean $_{post\text{-}treat}$ (HbA1c/weight/SBP/TC); SD of treatment effect on HbA1c/weight/SBP/TC = SQRT (SD $_{pre\text{-}treat}$ ^2 + SD $_{post\text{-}treat}$^2 - 2*0.4*SD $_{pre\text{-}treat}$ *SD $_{post\text{-}treat}$).
